# Consequences of Inhibiting Amyloid Precursor Protein Processing Enzymes on Synaptic Function and Plasticity

**DOI:** 10.1155/2012/272374

**Published:** 2012-06-26

**Authors:** Hui Wang, Andrea Megill, Kaiwen He, Alfredo Kirkwood, Hey-Kyoung Lee

**Affiliations:** ^1^Department of Biology, University of Maryland, College Park, MD 20742, USA; ^2^The Solomon H. Snyder Department of Neuroscience, The Zanvyl-Krieger Mind/Brain Institute, Johns Hopkins University, Baltimore, MD 21218, USA

## Abstract

Alzheimer's disease (AD) is a neurodegenerative disease, one of whose major pathological hallmarks is the accumulation of amyloid plaques comprised of aggregated **β**-amyloid (A**β**) peptides. It is now recognized that soluble A**β** oligomers may lead to synaptic dysfunctions early in AD pathology preceding plaque deposition. A**β** is produced by a sequential cleavage of amyloid precursor protein (APP) by the activity of **β**- and **γ**-secretases, which have been identified as major candidate therapeutic targets of AD. This paper focuses on how A**β** alters synaptic function and the functional consequences of inhibiting the activity of the two secretases responsible for A**β** generation. Abnormalities in synaptic function resulting from the absence or inhibition of the A**β**-producing enzymes suggest that A**β** itself may have normal physiological functions which are disrupted by abnormal accumulation of A**β** during AD pathology. This interpretation suggests that AD therapeutics targeting the **β**- and **γ**-secretases should be developed to restore normal levels of A**β** or combined with measures to circumvent the associated synaptic dysfunction(s) in order to have minimal impact on normal synaptic function.

## 1. Introduction

**Figure 1:**
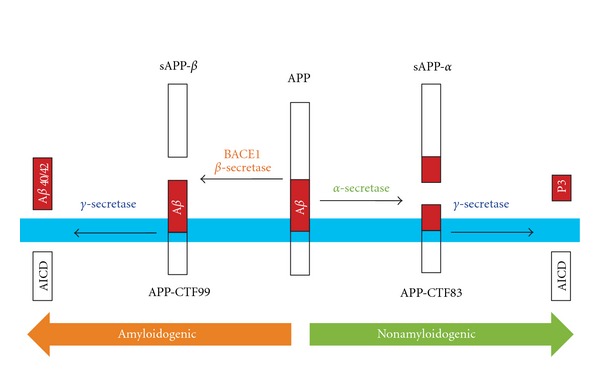
A diagram of amyloid precursor protein (APP) processing pathways. The transmembrane protein APP (membrane indicated in blue) can be processed by two pathways, the nonamyloidogenic *α*-secretase pathway and the amyloidogenic *β*-secretase pathway. In the nonamyloidogenic pathway, *α*-secretase cleaves in the middle of the *β*-amyloid (A*β*) region (red) to release the soluble APP-fragment sAPP-*α*. The APP C-terminal fragment 83 (APP-CTF83) is then cleaved by *γ*-secretase to release the APP intracellular domain (AICD) and P3 fragment. In the amyloidogenic pathway, *β*-secretase cleaves APP to produce the soluble fragment sAPP-*β*. APP-CTF99 is then cleaved by *γ*-secretase to produce A*β*_40_, A*β*_42_ and AICD.

Alzheimer's disease (AD) is a progressive neurodegenerative disorder, causing loss of synaptic contacts and cognitive decline. It is widely believed that AD is initiated by synaptic dysfunction, which may be the basis for memory loss in early stages of the disease [1, 2]. Current theories implicate the production of amyloid beta (A*β*) as a key molecular event that ultimately leads to neuronal degeneration and the clinical pathology seen in AD [3]. A*β* is produced by sequential proteolytic cleavage of amyloid precursor protein (APP) by two endoproteolytic enzymes, *β*- and *γ*-secretase (Figure 1). Therefore, inhibiting the activity of these enzymes has surfaced as one of the major disease-modifying approaches for AD [4]. However, in order to develop effective therapeutics, a detailed molecular and cellular understanding of the role of both secretases in synaptic function is necessary. In addition, since accumulating evidence suggests that the initial pathology of AD is a result of synaptic dysfunction [1, 2], understanding how A*β* production alters normal synaptic function and what types of synaptic functions are differentially affected by A*β* becomes important in developing effective therapeutics for disease intervention. In this paper, we will summarize a number of experimental observations that address how A*β* affects synaptic function, and review data obtained from genetically altered mice developed to test the feasibility of blocking APP-processing enzymes which unveiled functional roles for these enzymes in normal synaptic transmission and plasticity. We will also discuss a body of work, which investigates how synaptic function is affected by currently available therapies that target APP-processing enzymes. Before that we will briefly introduce the topic and current understanding of synaptic plasticity, which are relevant for the later discussions. 

## 2. Synaptic Plasticity and Memory Formation

It is widely believed that long-term changes in the strength of synaptic transmission underlie the formation of memories. Hebb is often recognized as the first person to crystallize this idea by proposing that coincident activity of pre- and postsynaptic neurons strengthens synaptic connections [5]. It was subsequently recognized that uncorrelated activity between two neurons should decrease the strength of synaptic transmission between them [6]. The strengthening of synaptic connections is termed long-term potentiation (LTP) and is experimentally produced by high-frequency stimulation [7], while the weakening of synaptic connections, produced by low-frequency stimulation [8, 9], is called long-term depression (LTD). Since their initial discovery, both LTP and LTD have been found to occur in a diverse set of synapses across many different brain areas (reviewed in [10]). These long lasting forms of synaptic plasticity share similar mechanisms of induction, expression, and maintenance with those of long-term consolidation of several forms of memory [11–19]. Moreover, long-term alterations in synaptic transmission, similar to characteristics of LTP and LTD, have been observed *in vivo* during various learning paradigms [20–24], which further suggests that LTP and LTD may be cellular substrates for memory formation.

While LTP and LTD are effective models for mediating synapse-specific changes required for memory formation, theoretical considerations indicate that maintaining the stability of the nervous system requires additional homeostatic plasticity mechanisms that operate at a slower time scale (hours to days) [25–29]. For example, without homeostatic regulation, the increase in postsynaptic activity after LTP might result in a vicious cycle of potentiation that not only degrades the capacity of neural circuits to store specific information but could also culminate in a run-away excitation of the neural network. There are several mechanisms of homeostasis that can stabilize the nervous system: adjusting excitatory synaptic transmission postsynaptically [26–30], modulating the excitability of neurons [31–33], changing inhibitory circuits [33–36], and altering presynaptic function [37–39]. While most studies of synaptic plasticity related to memory formation focus on LTP and LTD, it is prudent to understand that alterations in homeostatic plasticity can also affect learning and memory. 

## 3. Molecular Mechanisms of Synaptic Plasticity: A Brief Overview

While LTP and LTD have been observed in many different brain areas, the majority of knowledge about their molecular mechanisms comes from studies in the hippocampus. This is partly because the hippocampus is an area of the brain that is critically involved in the formation of long-term memories (reviewed in [16]). In addition, the hippocampus is one of the areas highly susceptible to amyloid pathology in most AD brains (reviewed in [2]). Therefore, we will briefly review the mechanisms of synaptic plasticity in the hippocampus.

In the hippocampus, two major forms of LTP and LTD are observed: one that is dependent on NMDA receptor (NMDAR) activation and another that is independent of NMDARs [16, 40]. The most widely studied forms of LTP and LTD are those dependent on NMDARs in the CA1 region; hence, their mechanisms have been fairly well characterized. Therefore, most of our discussion will focus on the NMDAR-dependent forms of LTP and LTD. NMDARs, due to activity-dependent relief of their Mg^2+^ block [41], act as coincident detectors for pre- and postsynaptic activity. In addition, activation of NMDARs allows influx of Ca^2+^ [42–44], which can act as a second messenger to activate various downstream effectors in the postsynaptic neuron. It is thought that both the magnitude and temporal pattern of Ca^2+^ increase determine the expression of either LTP or LTD, by differentially regulating the activity of protein kinases and phosphatases [15]. One of the key downstream events of LTP and LTD is the regulation of synaptic AMPA receptors (AMPARs) (for review see [45, 46]). AMPARs are the major mediators of fast excitatory synaptic transmission in the central nervous system (CNS); therefore their function directly dictates synaptic strength. Several studies demonstrated that LTP increases the synaptic content of AMPARs, predominantly by an activity-dependent insertion of receptors containing the GluA1 subunit (GluR1) [47–49]. This requires concomitant activation of Ca^2+^/calmodulin-dependent protein kinase II (CaMKII) and phosphorylation of the AMPAR subunit GluA1 at serine 818 (S818) [50] and serine 845 (S845) [51]. GluA1-S818 is a protein kinase C (PKC) phosphorylation site [50] while GluA1-S845 is a protein kinase A (PKA) phosphorylation site [52]. In addition to these two sites, phosphorylation of GluA1-S831, which can be phosphorylated by both PKC [52] and CaMKII [53, 54], has been shown to correlate with LTP [55, 56]. However, this site is not necessary for LTP [57] nor synaptic trafficking of AMPARs [47]. Many studies confirm that CaMKII, PKC, and PKA are involved in NMDAR-dependent LTP (reviewed in [46]). Consistent with a dominant role for GluA1 in mediating synaptic potentiation, GluA1 knockout mice [58], as well as mice lacking specific phosphorylation sites on GluA1 [59], display LTP deficits. On the other hand, NMDAR-dependent LTD is associated with an activity-dependent removal of synaptic AMPARs [60]. This process depends on endocytosis of GluA2-containing receptors [61–67] but also requires dephosphorylation at GluA1-S845 [56, 59, 68].

While regulation of synaptic AMPARs, through synaptic targeting and phosphorylation, is involved in the initial expression of LTP and LTD, maintenance of these forms of plasticity involves additional mechanisms. Collectively, data from many studies report that blocking new protein synthesis inhibits the late phase of long-term synaptic plasticity [69–74]. This parallels the requirement for new protein synthesis in the formation of long-term memory in intact animals [75, 76] (see review [77]). Transcriptional activation is also necessary for the maintenance of some forms of long-term synaptic plasticity [78]. So far, it is known that multiple transcription factors are activated immediately after induction of LTP. Increased transcription of several immediate early genes (IEG) is especially important [79] since they enhance new protein synthesis [12, 16]. Interestingly, some, if not all, of these transcriptional regulators are also required for long-term memory formation. Disruption of cAMP response element-binding protein (CREB) levels, a Ca^2+^-dependent transcription factor, in either the hippocampus or the amygdala has been found to impair specific long-term memory but not initial acquisition or short-term memory formation [80–82]. Inhibiting the expression of Arc/Arg 3.1 (activity-regulated cytoskeletal protein/activity-regulated gene 3.1), an IEG, in the hippocampus also impairs long-term memory consolidation [83]. 

## 4. Exogenous A***β*** Application Alters Synaptic Function

Much of the molecular understanding of AD came from studying familial AD (FAD-) linked mutations, which have been found in genes encoding APP and presenilin 1 and 2 (PS1 and 2) in AD patients. These mutations are linked to elevated A*β* production [84, 85]. This is because many FAD-linked mutations make APP a more favorable substrate for the amyloidogenic cleavage pathway leading to increased A*β* production. Since FAD patients often harbor multiple mutations, many of the AD mouse models also carry several FAD mutations. However, depending on the combination of the mutations and their variants, distinct phenotypes are observed across age and brain regions studied (for an extensive recent review on electrophysiological studies of various AD transgenic (Tg) mouse models see [86]).

Although different AD mouse models show deficits in synaptic function, it cannot be taken for granted that these deficits are caused directly by the enhanced production of A*β* peptides (especially A*β*_42_, which is the major component of extracellular senile plaques). In order to directly test the role of A*β* in altering synaptic function, many studies have investigated synaptic properties and synaptic plasticity following exogenous application of various A*β* peptides. 

*In vitro* studies done in either the medial perforant path to dentate granule cells or the Schaffer collateral inputs to CA1 neurons reported that application of subneurotoxic concentrations of A*β* peptides (i.e., A*β*_42_, A*β*_40_, or A*β*_25–35_) inhibit LTP induction without affecting basal synaptic transmission [87–89]. A similar result was found in an *in vivo* study, where naturally secreted A*β* collected from cells expressing mutated APP (V717F mutation in APP_751_) was injected into the CA1 region of hippocampus which prevented stable LTP maintenance [90]. This study further showed that soluble A*β* oligomers, not monomeric A*β*, or A*β* fibrils, are responsible for blocking LTP [90]. In addition, *in vivo* injection of A*β* peptides (i.e., A*β*_42_ or the C-terminal of APP which contains the A*β* fragment) is reported to facilitate LTD and LTP reversal (called depotentiation) in the CA1 region [91]. A majority of studies suggest that while fibrillar A*β* accumulation is found in senile plaques that are a hallmark of AD, it is the soluble A*β* oligomers that disturb synaptic function and lead to neurodegeneration in AD [90, 92]. 

### 4.1. Postsynaptic Alterations by A*β*

Soluble A*β* oligomers in AD brains have been found to bind to neuronal surfaces [93], specifically to a subset of synapses where they colocalize with a postsynaptic density marker PSD95 [94], suggesting that A*β* may regulate postsynaptic function directly. One candidate target of A*β* is NMDARs. It was found that synthetic A*β*_40_ peptides can selectively augment NMDAR current, without affecting AMPAR current, in the dentate gyrus of acute hippocampal slices [95]. Consistent with this, APP_Ind_ (V717F mutation) Tg mice show an enhancement in the ratio of NMDAR-to-AMPAR-mediated synaptic transmission in the CA1 region [96]. However, contradictory results are reported from later studies. A recent study showed that application of both synthetic A*β*_42_ peptides and naturally secreted A*β*, from APP_Swe_ (K670N/M671L mutation) Tg mice, promotes endocytosis of surface NMDARs and hence depresses NMDAR current in wildtype cultured cortical neurons [97]. Moreover, they also found reduced surface expression of NMDARs in cultured cortical neurons from APP_Swe_ Tg mice [97]. Other studies found downregulation of surface AMPARs in neurons overexpressing either wildtype or APP_Swe_ or when wildtype neurons were treated with exogenous A*β*_42_ peptides [98, 99]. This is mediated not only by endocytosis of synaptic AMPARs via mechanisms shared by LTD [99] but also through a reduction in basal levels of GluA1-S845 phosphorylation by activating the calcium-dependent phosphatase, calcineurin, as well as interrupting extrasynaptic delivery of AMPARs [100]. Contradictory results on the effects of A*β* on AMPAR and NMDAR regulation may be due to several variables. First, there is evidence that A*β*_40_ and A*β*_42_ peptides may have distinct functions in AD pathology. For example, a majority of FAD-linked PS1 mutations cause a reduction in *A*β*_40_* peptides and therefore an increase in the A*β*_42_/A*β*_40_ ratio [101, 102]. Second, there are differences in experimental preparations. Both Wu et al. [95] and Hsia et al. [96] were working with acute adult hippocampal slices, while Snyder et al. [97], Almeida et al. [98], Hsieh et al. [99], and Miñano-Molina et al. [100] were using either cultured neurons from embryonic mice or organotypic hippocampal slice cultures prepared from early postnatal mice. Third, the presence or absence of APP itself may have also affected the results. Indeed there is evidence that uncleaved full-length APP may promote synapse formation and enhance excitatory synaptic function (see [103] for a recent review).

In any case, A*β*-mediated alterations in NMDAR function suggest that A*β* will affect downstream Ca^2+^-dependent signaling pathways. Calcineurin, a Ca^2+^-activated protein phosphatase, may be one of the downstream signaling molecules affected by A*β*, since it is required for the inhibition of perforant pathway LTP [88], endocytosis of surface AMPARs [99], as well as dephosphorylation of GluA1-S845 [100]. In addition to activating calcineurin, A*β* prevents the activation of CaMKII, a Ca^2+^-dependent protein kinase necessary for LTP, and decreases the synaptic clustering of CaMKII, which correlates with a reduction in the phosphorylation of GluA1-S831, surface expression of GluA1, and AMPAR-mediated EPSCs [89, 104]. Together, these data are consistent with the idea that A*β* oligomers impair LTP and facilitate LTD [56, 105, 106]. 

A*β* has also been found to modify regulation of gene expression. A*β* peptides have been found to alter CREB signaling, causing synaptic dysfunction and memory deficits (reviewed in [107]). In addition, treating cultured hippocampal neurons with soluble A*β* oligomers induces rapid expression of the IEG Arc/Arg 3.1 [94], which is implicated in synaptic plasticity [83, 108, 109]. Because overexpression of Arc/Arg 3.1 causes learning dysfunction [110], possibly via reducing surface expression of GluA1-containing AMPARs [109], this would suggest that A*β* oligomer-induced Arc/Arg 3.1 expression may in fact interfere with normal synaptic plasticity. However, this study is seemingly at odds with the results of Echeverria and colleagues, which reported a strong inhibition of BDNF-induced increase in Arc expression in cultured cortical neurons treated with A*β* oligomers [111]. Similarly, there is also a report that synaptic plasticity-related genes, including Arc/Arg 3.1, are reduced in transgenic mice expressing FAD-linked mutations in APP and PS1 [112]. The apparent differences in Arc expression caused by A*β* could be due to different experimental systems or to the differential effects of different concentrations of A*β* oligomers.

### 4.2. Presynaptic Alterations by A*β*

Besides influencing postsynaptic function, A*β* is also implicated in presynaptic modifications. A recent study reported that 8 nM A*β*_42_ globulomer (a highly stable globular oligomeric A*β*) could directly inhibit presynaptic P/Q type Ca^2+^ channels and decrease vesicle release [113]. Moreover, application of synthetic A*β* to cultured hippocampal neurons causes a downregulation of dynamin, a protein critical for synaptic vesicle endocytosis, and interrupts synaptic vesicle recycling [114, 115]. This result is consistent with the observed reduction in dynamin levels in human AD brains [116]. These findings may explain the observation that A*β*_42_ globulomer causes a decrease in basal synaptic transmission at the Schaffer collateral to CA1 synapses in hippocampal slice culture [117]. Recently, Kelly et al. reported that the reduction in dynamin is dependent on Ca^2+^ influx through activated NMDARs as well as activation of a calcium-activated intracellular cysteine protease calpain [114, 118]. These results not only suggest that there may be retrograde signaling from postsynaptic to presynaptic terminals but also establish an interesting relationship between A*β*, NMDARs, and calpain. It has been found that A*β*_42_ peptides can activate calpain-mediated cleavage of p35 to p25 [119], which then upregulates mRNA and protein expression of *β*-secretase (BACE1) [120, 121], a critical enzyme for A*β* formation (discussed in the following sections). This indicates that there is a positive feedback between A*β* production and calpain activation. Calpain inhibitors can fully prevent deficits in basal synaptic transmission caused by A*β* globulomer application in hippocampal slice culture to a comparable level as using an NMDAR antagonist memantine [117]. This suggests that A*β* acts through NMDARs and calpain: a potential signaling cascade being NMDAR-medicated Ca^2+^ influx activating intracellular calpain, which then promotes p25/cdk5-dependent transcription of downstream genes, including BACE1 [120]. 

### 4.3. Other Targets of A*β* That Affect Synaptic Plasticity

Recent studies suggest that the *α*7-nicotinic acetylcholine receptor (*α*7-nAChR), a Ca^2+^-permeable homopentameric ion channel highly expressed in the hippocampus and cerebral cortex [122], is another potential target of A*β*. High affinity binding between A*β*_42_ peptides and *α*7-nAChRs [123, 124] either inhibits [125–128] or activates *α*7-nAChR signaling [129]. It is possible that A*β*_42_ peptides may facilitate *α*7-nAChRs at low concentrations but may inhibit *α*7-nAChRs when the burden of A*β* increases [129, 130]. This concentration-dependent role of A*β* peptides is suggested from studies showing that at normal concentrations (picomolar range), A*β* peptides positively regulate presynaptic release at hippocampal synapses and facilitate CA1 LTP and learning by activating *α*7-nAChRs, whereas when the level of A*β* is low or high (nanomolar range), A*β* peptides cause either deficits in presynaptic function or abolish hippocampal LTP and learning via its interaction with *α*7-nAChRs [131–133].

**Figure 2:**
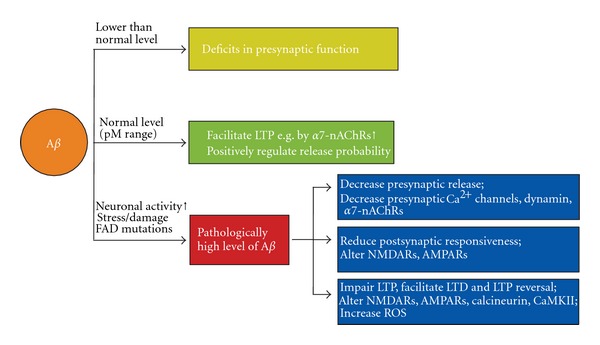
Concentration-dependent effects of A*β* on synaptic function. At normal physiological levels (picomolar range), A*β* peptides have positive effects on synaptic function: they can positively regulate presynaptic release probability and facilitate learning and LTP in CA1 by activating *α*7-nAChRs. However, when the concentration of A*β* peptides is lower than normal, presynaptic function is impaired. On the other hand, under pathological conditions, such as increased neuronal activity, stress, or the presence of familial Alzheimer's disease (FAD) mutations, the increase in A*β* peptide concentration produces pathological effects, including decreased presynaptic neurotransmitter release, reduced postsynaptic responsiveness, LTP impairment, and LTD facilitation. Therefore, maintaining the concentration of A*β* peptides within a normal physiological range is essential and should be the goal for developing effective treatments for Alzheimer's disease.

Moreover, the concentration-dependent effect of A*β* is also reflected by its ability to regulate reactive oxygen species (ROS). ROS have been found to have physiological roles in maintaining normal synaptic plasticity. However, high levels of ROS have been found in both AD animal models and human patients, leading to oxidative damage related to AD pathology (reviewed in [134]). Recently, Ma and colleagues found that exogenous treatment of A*β*_42_ (500 nM) increased mitochondria superoxide, which they reported is a cause of synaptic dysfunction induced by A*β*. In particular, decreasing mitochondrial superoxide levels reversed A*β*-induced CA1 LTP impairments [135]. Given the normal physiological role of A*β* and ROS at intermediate levels, this finding suggests that ROS imbalance, caused by A*β* toxicity, may lead to synaptic dysfunction in AD. It also implies that A*β* levels exceeding the normal range may initiate the abnormalities in synaptic function (Figure 2). 

In summary, pathologically high levels of A*β* can disturb the ROS balance and interfere with both pre- and postsynaptic function, presumably by affecting NMDARs, presynaptic P/Q Ca^2+^ channels, and/or *α*7-nAChRs, thereby interrupting subsequent Ca^2+^ signaling leading to altered synaptic function.

## 5. Neuronal Activity Can Regulate APP Processing and A***β*** Levels

Data from both transgenic mice and exogenous A*β* application studies suggest that alterations in A*β* levels change neuronal activity and synaptic function. *In vivo* two-photon Ca^2+^ imaging of APP23xPS45 mice showed that cortical neurons near amyloid plaques are hyperactive, while the percentage of hypoactive cortical neurons is enhanced at locations further away from a plaque [136]. The disparate change in neuronal activity relative to the location of a neuron to amyloid plaques may reflect differences in local A*β* concentration. It is now evident that neuronal activity itself can also regulate APP-processing leading to alterations in A*β* production. In 1993, a study reported that electrical stimulation not only increases neurotransmitter release in rat hippocampal slices but also enhances the release of APP cleavage products [137]. In agreement with this finding, ten years later, Kamenetz and colleagues [138] found that neuronal activity can bidirectionally control A*β* levels in organotypic hippocampal slice cultures from APP_Swe_ Tg mice. Blocking neuronal activity in this preparation by tetrodotoxin (TTX) treatment reduced A*β* levels, while increasing neuronal activity with picrotoxin (PTX) enhanced A*β* secretion [138]. The experimental paradigm used by Kamenetz et al. to manipulate neuronal activity is reported to produce homeostatic synaptic plasticity termed “synaptic scaling” [28], which globally up- or downregulates all excitatory synapses following prolonged decrease or increase, respectively, in neuronal activity [29]. This suggests that A*β* may play a role in regulating homeostasis of excitatory synapses in normal brains. In addition, the cellular mechanism responsible for regulating APP-processing and A*β*production in response to neuronal activity is possibly through enhancing the accessibility of APP to *γ*-secretase cleavage [138] and/or depressing *γ*-secretase function [139]. It has recently been shown that PS1, the catalytic subunit of the *γ*-secretase complex, is necessary to scale up excitatory synapses following reduced network activity and that PS1 knockout mice show deficits in synaptic scaling [140]. Moreover, Wu and colleagues have reported that the immediate early gene Arc is required for the activity-dependent increase in A*β* production [141]. They found that Arc directly binds the N terminus of PS1 and plays an important role in trafficking the *γ*-secretase complex to early endosomes where APP is processed through the amyloidogenic pathway to produce A*β* peptides. In addition, Arc contributes to A*β* levels and plaque load in APP_Swe_; PS1ΔE9 mice and Arc expression are elevated in medial frontal cortex of AD patients [141]. These results provide a cellular mechanism coupling A*β* generation to neuronal activity and may explain why people who suffer from hypoxia, which usually causes an abnormal enhancement in neuronal activity [142], have a higher risk for developing AD [143]. 

Consistent with the idea that A*β* induces homeostatic adaptation to increases in activity, *in vivo* studies have also shown that either electrical stimulation or endogenous whisker activity proportionally regulates interstitial fluid (ISF) A*β* levels in Tg2576 mice, which overexpress human APP carrying the Swedish (K670N/M671L) mutation [144–146]. However, there are also contradictory results. Tampellini et al. have shown that synaptic activity decreases intracellular A*β* in primary neuronal culture, as well as in the barrel cortex of 4-month-old Tg19959 mice, which overexpress human APP carrying the Swedish (K670N/M671L) and Indiana (V717F) mutations [147], likely by enhancing A*β* degradation [148]. Zhang et al. have reported that prolonged olfactory deprivation facilitates amyloid plaque deposition in the olfactory bulb and piriform cortex of 7–24-month-old Tg2576 mice [149]. These contradictions may be due to age, region, and paradigm differences. Another possibility is that normal neuronal activity regulates A*β* levels by balancing A*β* release and degradation and that either hyperactivity or hypoactivity may break this balance leading to A*β* accumulation. 

## 6. Physiological Roles of APP and A***β***

Proteolytic processing of APP not only produces A*β* peptides but also other products. Some functions of these products have been identified (reviewed in [150]). For example, the cytoplasmic tail of APP, APP intracellular domain (AICD), is shown to participate in transcriptional regulation [151]. To evaluate other normal physiological roles of APP, mice lacking APP were generated. APP knockouts show enhanced excitatory synaptic activity and neurite growth [152], which is consistent with the finding that APP-deficient mice are more susceptible to glutamate-induced toxicity [153]. Similar to APP, A*β* peptides also have normal physiological functions. Normal levels (picomolar range) of A*β* peptides regulate synaptic function by positively increasing presynaptic release at hippocampal synapses and facilitating learning and LTP in CA1 [131–133]. Moreover, normal levels of A*β* may be essential for neurons, because preventing A*β* production by adding *β*- or *γ*-secretase inhibitors in cultured neurons causes cell death, which can be rescued by applying synthetic A*β* peptides to culture medium [154]. In addition, activity-dependent changes in A*β* may in fact play a role in maintaining homeostasis by acting as a negative feedback regulator of excitatory synaptic transmission [138]. 

Collectively, these data suggest that proteolytic processing of APP and the presence of a normal physiological dose of A*β* may be required for maintaining proper neuronal activity and brain function. While the therapeutic benefits of targeting APP-processing and A*β* production are still attractive, it should be noted that AD pathology is most likely triggered only when A*β* levels exceed the normal range and that the physiological processing of APP and A*β* production may be important in maintaining normal brain functions. Therefore, partial inhibition, but not complete blockade, of A*β* production might be a useful approach for AD therapeutics. A recent study supports this view. Immunizing APP_Ind_ Tg mice against A*β*, which lowered A*β* levels, decreased senile plaque formation and rescued loss of neuronal integrity seen previously in aged mice [155]. However, A*β*-immunotherapy in clinical trials reported severe complications, which must be overcome (for review articles on this topic please see [156–158]).

## 7. Role of BACE1 in Synaptic Function

As mentioned above, A*β* peptides are generated by sequential cleavage of APP by *β*- and *γ*-secretase (Figure 1). In the brain, beta-site APP cleaving enzyme (BACE1), a transmembrane aspartic protease, has been found to be the major neuronal *β*-secretase [159–162]. Mice lacking the BACE1 gene show no *β*-secretase activity and essentially no A*β* (A*β*_40_ and A*β*_42_) production in the brain compared to wildtype littermates. Initial characterization of BACE1 knockouts (BACE1^−/−^) showed that they are viable and fertile, with no gross differences in behavior or development [159–161, 163]. Furthermore, knocking out the BACE1 gene in mouse models of AD was able to rescue hippocampus-dependent memory deficits [163–165] and ameliorate impaired hippocampal cholinergic regulation of neuronal excitability [163]. These findings were quite encouraging and suggested that BACE1 may be a good therapeutic target for treating AD [4, 166, 167].

However, recent studies have found that BACE1 has normal physiological functions in synaptic transmission and plasticity in both CA1 and CA3 regions of the hippocampus (Table 1). Laird et al. found that BACE1^−/−^ mice display deficits in both synaptic transmission and plasticity at the hippocampal Schaffer collateral to CA1 synapses [164]. While BACE1^−/−^ mice display normal AMPAR- and NMDAR-mediated synaptic transmission, these synapses show a larger paired-pulse facilitation (PPF) ratio compared to wildtype littermates when tested with paired-pulse stimuli at a 50 ms interstimulus interval [164]. Changes in PPF ratio are linked to alterations in presynaptic function [168]. Therefore, the increase in PPF ratio observed in BACE1^−/−^ mice indicates a reduction in presynaptic function, which is consistent with the high expression of BACE1 in presynaptic terminals [164]. In addition to reflecting presynaptic changes, recent data suggest that alterations in PPF ratio can also be caused by postsynaptic modifications, such as by varying the subunit composition of AMPARs [169]. Therefore, it is possible that knockout of BACE1 may also affect postsynaptic AMPAR function. Besides alterations in PPF ratio, BACE1^−/−^ mice also showed a larger dedepression (reversal of LTD) induced by high frequency theta burst stimulation (TBS) at the Schaffer collateral inputs to CA1 [164]. In contrast, the same TBS protocol-induced LTP remained unchanged [164]. As LTP and dedepression have separate underlying mechanisms [56], these data suggest BACE1 may play a regulatory role in the dedepression pathway, while not affecting the mechanisms that lead to LTP. Laird and colleagues also found evidence that the enhanced dedepression is due to larger summation of responses during TBS, specifically following LTD induction. Enhanced summation of synaptic responses during the induction of de-depression despite normal basal synaptic transmission suggests that BACE1 may play a specific role in activity-dependent high-frequency information transfer across synapses. Also, the abnormal increase in the magnitude of de-depression reflects that LTD expression may be easily disrupted when knocking out BACE1, which could interfere with memory formation and storage. Consistent with this interpretation, detailed behavioral studies of BACE1^−/−^ mice reported problems in both cognitive and emotional memory tests [164, 170, 171].

Although the majority of studies characterizing synaptic function of BACE1^−/−^ mice have been performed in the CA1 region of the hippocampus [163, 164, 171], the expression of BACE1 is most prominent in the mossy fiber terminals that synapse onto CA3 pyramidal neurons [164, 172]. Recently, we reported that BACE1^−/−^ mice display severe deficits in presynaptic function at these synapses, including a reduction in presynaptic release and an absence of mossy fiber LTP, which is normally expressed by a long-term increase in presynaptic release [173]. Moreover, BACE1^−/−^ mice exhibited a slightly larger mossy fiber LTD, which could not be reversed [174]. These results suggest that BACE1 function is crucial for normal synaptic transmission and activity-dependent presynaptic potentiation at these synapses. We further found evidence that the presynaptic dysfunction in BACE1^−/−^ mice is likely at the level of presynaptic Ca^2+^ signaling, because the mossy fiber LTP deficit in BACE1^−/−^ mice could be recovered by increasing the extracellular Ca^2+^ concentration. This suggests that the signaling downstream of Ca^2+^ is more or less intact in BACE1^−/−^ mice, which was confirmed by the fact that the magnitude of presynaptic potentiation resulting from direct activation of the cAMP signaling pathway is normal in BACE1^−/−^ mice [174]. Therefore, it is possible that manipulations that enhance presynaptic Ca^2+^ may overcome the synaptic deficits caused by inhibiting BACE1 activity. In line with this, we recently showed that activation of Ca^2+^-permeable *α*7-nAChRs, by nicotine or *α*7-nAChRs agonist, can restore PPF ratio and mossy fiber LTP in BACE1^−/−^ mice [175]. The cellular mechanism of nicotine-induced rescue is dependent on the recruitment of Ca^2+^-induced Ca^2+^-release (CICR) from intracellular Ca^2+^ stores through ryanodine receptors [175]. These results suggest that nicotine and *α*7-nAChR agonists may be a potential pharmacological means to circumvent the synaptic dysfunctions caused by BACE1 inhibition.

**Figure 3:**
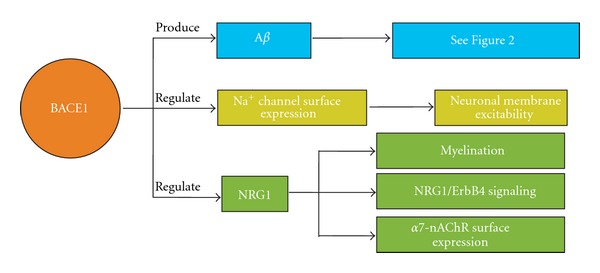
The roles of BACE1 in synaptic function. Besides cleaving APP to produce A*β* peptides, BACE1 has been found to have other substrates. It can process the *β*2 subunit of the voltage-gated sodium (Na^+^) channels, which can regulate Na^+^ channel surface expression and in turn modulate neuronal excitability. In addition, BACE1 can cleave NRG1, which plays a crucial role in myelination and NRG1/ErbB4 signaling. Recently, it has been showed that NRG1 can regulate cell surface expression of *α*7-nAChRs, which can also affect synaptic transmission.

Since synaptic deficits are seen in both the CA1 and CA3 regions of BACE1^−/−^ mice, it indicates that BACE1 may play a general role in regulating presynaptic function. Reduced A*β* levels have been shown to produce deficits in presynaptic function [131], which may explain the synaptic phenotype seen in BACE1^−/−^ mice. However, whether presynaptic deficits in BACE1^−/−^ mice are solely due to a lack of APP-processing is unclear. An alternative possibility is that the synaptic dysfunction seen in BACE1^−/−^ mice may arise from abnormal processing of substrates other than APP (Figure 3).

It has been shown that the auxiliary *β*2 subunit of the voltage-gated sodium channel (Na_v_1) is a substrate of BACE1 [186, 187]. The *β*2 subunit of the Na_v_1 channel is important for plasma membrane expression of functional Na^+^ channels, which are critical for generating action potentials. Among the ten different types of Na_v_1 channels, Na_v_1.1, Na_v_1.2, Na_v_1.3, and Na_v_1.6 are expressed mainly in the central nervous system (CNS) [188]. BACE1 regulates the surface expression of these types of Na_v_1 channels by cleaving the *β*2 subunit. In transgenic mice overexpressing BACE1, there is an increase in the Na_v_1.1 *α*-subunit mRNA and protein levels, but a decrease in the surface expression of functional Na_v_1.1 channels due to cleavage of the *β*2 subunits [187, 189]. The interpretation is that the full-length *β*2 subunit promotes surface expression of Na_v_1.1 channels, but the *β*2-intracellular domain (ICD), which is produced by a sequential cleavage by BACE1 and *γ*-secretase, increases transcription of the Na_v_1.1 *α*-subunit gene. Consistent with this, BACE1^−/−^ mice display a decrease in Na_v_1.1 *α*-subunit mRNA and protein [190]. However, there is a compensatory increase in the surface expression of Na_v_1.2 in BACE1^−/−^ mice, which correlates with the hyperexcitability and seizure phenotypes seen in these mice [191]. These results suggest that the ability of BACE1 to regulate the Na_v_1 family of Na^+^ channels is rather complex but suggest a role for BACE1 in regulating neuronal excitability. 

Another candidate substrate for BACE1 is neuregulin-1 (NRG1), which is an axonal signaling molecule critical for regulating myelination [192]. Willem and colleagues found that BACE1^−/−^ mice show hypomyelination in the peripheral nerves [193], while another study detected loss of myelination in the central nerves [194]. Both of these studies showed an accumulation of unprocessed NRG1 and a reduction in its cleavage products, suggesting that NRG1 is a potential substrate for BACE1 cleavage and that this process is important for myelination of axons [193, 194]. Recently, it has been shown that the absence of NRG1 processing in BACE1^−/−^ mice decreased postsynaptic function of ErbB4, a receptor for NRG1 [195]. NRG1/ErbB4 signaling has been suggested to regulate synaptic function and plasticity, mainly via regulation of postsynaptic glutamate receptors [196–198]. Additionally, abnormal processing of NRG1 may also affect presynaptic release by regulating the expression of *α*7-nAChRs [199, 200] which allows Ca^2+^ influx [122]. Indeed, presynaptic nAChRs can increase glutamate release [201–203], likely via the *α*7 containing nAChRs [204]. These results suggest that a lack of NRG1 cleavage resulting from BACE1 inhibition can alter synaptic function both pre- and postsynaptically.

Accumulating data on the biological roles of BACE1, particularly evidence that completes inhibition of BACE1 activity which is deleterious for normal neuronal function, suggests caution for using BACE1 inhibitors as a treatment for AD. In order to improve the development of effective therapeutics that target this enzyme, we need to identify ways to avoid the synaptic dysfunction associated with blocking BACE1, which may include partial inhibition strategies. 

### 7.1. Partial Inhibition or Conditional Knockdown of BACE1

It has been shown that A*β* burden is dose dependent on BACE1 activity; therefore, partial inhibition or conditional knockdown of BACE1 may be beneficial for AD treatment. To test this, Kimura and colleagues crossed BACE1 heterozygous mice with a line of transgenic mice carrying a combination of 5 FAD-linked mutations in human APP and PS1 (5XFAD); they found that partial reduction of BACE1 improved remote and recent memory and restored CA1 LTP [176]. Researchers have also successfully suppressed BACE1 activity by using RNA interference (RNAi) *in vitro* [205, 206] and *in  vivo* [164, 207]. Lentiviral BACE1 siRNA delivered into the hippocampus has been found to effectively reduce A*β* production, neurodegeneration, and behavioral deficits in APP transgenic mice [164, 207]. Characterizing synaptic function in the BACE1 siRNA knockdown models may provide information about acute effects of blocking BACE1 function. In addition, siRNA knockdown of BACE1 in APP transgenic lines will better approximate clinical situations, hence allowing us to better estimate the feasibility of developing an effective treatment for AD by BACE1 inhibition. 

### 7.2. BACE1 Inhibitors

 Since the identification of BACE1, the development of BACE1 inhibitors has been initiated. However, the progress was slow, probably due to the difficulty of identifying small molecules that can pass through the blood brain barrier and also have high stability and good pharmaceutical properties [208, 209]. So far, several BACE1 inhibitors have been discovered; among them only CTS-21166 has passed Phase I clinical trials (see review [208, 210]). Many BACE1 inhibitors have been shown to decrease soluble A*β* production, amyloid plaque deposition, as well as improve cognitive function in AD animal models [211–216]. Surprisingly, none of them have been tested to determine their ability to improve synaptic dysfunction, the cellular mechanism that correlates with cognitive decline. A critical question is whether these inhibitors can recover synaptic deficits seen in AD models or whether they may produce additional defects as seen in BACE1^−/−^ mice. 

### 7.3. Transcriptional and miRNA Regulation of BACE1 

There are several reports of transcriptional regulation of BACE1. Nie et al. have shown that activation of *α*4*β*2 nAChR can decrease BACE1 transcription through the ERK1-NF*κ*B pathway *in vitro* [217]; Wen and colleagues reported that overexpression of p25, an activator of cdk5, can increase BACE1 mRNA and protein levels likely through interactions of signal transducer and activator of transcription (STAT3) with the BACE1 promoter [120]. In addition, in the brains of sporadic AD patients, an increase in BACE1 levels is correlated with a decrease in a subset of microRNAs (miRNA), especially the miR-29a/b-1 miRNA cluster [218]. miRNAs regulate mRNA translation. Therefore, it is possible that an increase in specific miRNA levels can downregulate BACE1 protein expression and decrease A*β* burden. These findings provide various ways to regulate BACE1 expression.

### 7.4. Endogenous BACE1 Activity Modulators

Recently, studies have shown that during sporadic AD or in AD animal models, the activities of certain endogenous molecules are modified, causing an increase in BACE1 activity. For example, sphingosine-1-phosphate (S1P) phosphorylation of the translation initiation factor eIF2*α* and calpain activity are increased in AD, which can lead to an increase in BACE1 activity [117, 121, 219–221]. On the other hand, decreased activity in conjugated linoleic acid (CLA), acetylcholinesterase inhibitor galantamine (Gal), copper chaperone for superoxide dismutase (CCS), PPAR*γ* coactivator-1*α* (PGC-1*α*), the trafficking molecule GGA3, as well as Fbx2-E3 ligase during AD can lead to increased BACE1 protein levels [177, 222–228]. So far, only the effect of Fbx2 on synaptic plasticity has been tested. Adenoviral-Fbx2 transfection significantly improves CA1 LTP in Tg2576 mice without affecting basal synaptic transmission [177]. While these molecules may be potential targets for controlling BACE1 activity, further studies need to verify whether synaptic function can be improved by manipulating the activity of these BACE1 modulators.

## 8. Presenilin: Its Physiological Roles and Relationship with Alzheimer's Disease

Presenilin 1 (PS1) is the catalytic component of the *γ*-secretase complex. Following BACE1 cleavage, *γ*-secretase cleaves the transmembrane domain of APP, releasing A*β* peptides (Figure 1). The active *γ*-secretase complex is composed of four different proteins, all of which are required for the protease to function (for a good review on the composition of *γ*-secretase, see [229]); however, PS1 receives the most attention stemming from its identification as the major locus for early onset FAD [230]. Since the accumulation and deposition of extracellular A*β* have been emphasized in the progression of AD [92], the identification of several FAD-linked mutations in PS1 led to many studies investigating how dysfunction of this protein contributes to AD. FAD-linked mutations in PS1 facilitate the production of the more pathogenic A*β*_42_ peptide [85, 101], which is the major constituent of senile plaques found in the brains of AD patients. Here, we will briefly summarize the functions of presenilins and focus on how they play a role in normal synaptic regulation and also during AD. Key points are summarized in Table 2.

To investigate the normal physiological functions of PS1, many genetic knockout experiments have been conducted. Knockout of PS1 causes abnormal development and perinatal death [231–235]. FAD-linked mutations have also been discovered in Presenilin 2 (PS2), which is highly similar to PS1 in both sequence and structure [236]; however, PS2 knockout mice are viable and fertile with only mild age-dependent pulmonary fibrosis and hemorrhage [237]. This suggests PS1 is sufficient to maintain the majority of regular physiological activities and that these two homologs share little overlapping function. Another study using PS1^+/−^; PS2^−/−^ mice found that they could live normally until 6 months of age, after which most developed an autoimmune disease and benign skin hyperplasia [238]. The lethal effect of knocking out PS1 is not surprising considering that *γ*-secretase is involved in the processing of many other substrates beside APP [239–241], one of the most important being the Notch receptor, a protein that is critical in cell differentiation during embryonic development [231, 239, 240, 242]. 

*γ*-secretase still remains to be a promising candidate for AD drug targets because it is thought that the function of PS1 might not be as critical in the adult brain, unlike during embryonic development, and/or partial inhibition of the enzymatic activity may still be feasible. Encouragingly, mice with conditional knockout (cKO) of PS1, in which PS1 expression was eliminated in most neurons of the cerebral cortex in the postnatal brain, were viable and had nearly normal phenotypes, including normal basal synaptic transmission and plasticity, with only mild deficits in long-term spatial memory [178, 179]. A*β*_40_ and A*β*_42_ levels were also reduced in the cortex of PS1 cKO mice, providing evidence in support of targeting PS1 as a potential antiamyloid therapy in AD. Another promising finding was that regulation of Notch activity in the adult brain was unaffected and independent of PS1, contrasting the dependency of Notch signaling during embryonic brain development. This suggests PS2 may be able to compensate for the loss of PS1 in the adult brain and leads one to question whether knockout of both PS1 and PS2 will lead to more extreme deficits. To test this hypothesis, Saura and colleagues [179] generated forebrain-specific PS1/PS2 conditional double knockout (PS cDKO) mice. These mice exhibit cognitive impairments as well as deficits in hippocampal synaptic plasticity, which appear earlier than in the PS1 cKO mice. PS cDKO mice also developed age-dependent and progressive neurodegeneration, including loss of dendritic spines and presynaptic terminals [179]. Together, this suggests that in the adult brain the role of PS1 in regulating Notch signaling may not be as important but that presenilins are required for normal hippocampal synaptic plasticity, memory formation, and age-dependent neuronal survival.

It is encouraging that conditional inactivation of PS1 is able to decrease A*β* levels in the adult brain without effecting Notch signaling [178]. In order to examine the possibility of using inactivation of PS1 as a therapy for AD, PS1 cKO mice have been crossed with transgenic mice expressing different FAD-linked mutations in APP. The first study developed postnatal neuron-specific inactivation of PS1 (PS1^−/−^) in transgenic mice overexpressing human APP with the London mutation (V717I), APPxPS1^(−/−)^ [243]. This group had previously shown that APP(V717I) mice had increased levels of A*β*_42_ peptides as early as 2 months, leading to plaque development at 13 months old [244], as well as cognitive impairment and reduced hippocampal LTP. APPxPS1^(−/−)^ mice showed reduced A*β* and amyloid plaque formation, even at 18 months. While hippocampal CA1 LTP was rescued in APPxPS1^(−/−)^ mice, they still showed impaired cognition. A second study used the forebrain-specific PS1 cKO mice, mentioned previously [178, 179], to inactivate PS1 in an APP transgenic that overexpressed human APP containing the Swedish (K670N/M671L) and Indiana (V717F) mutations to generate PS1 cKO;APP Tg [183]. Similar to the previous study, these mice also had reduced amyloid phenotypes compared to APP Tg mice, but there was still no long-term improvement in cognitive function. Conditional inactivation of PS1 was only able to rescue learning and memory deficits seen in young but not old mice [183]. Together, these data indicate a causative role for A*β* peptides in LTP deficits and demonstrate that inactivation of PS1 in Tg mice can decrease the amyloid pathology of AD and restore LTP impairments in young mice. One question elicited from the above studies is that, if conditional knockout of PS1 is able to reduce amyloid pathologies and rescue certain LTP impairments, why is it not able to rescue cognitive deficits seen in these AD mice and why is it not able to sustain LTP improvements in older mice? One possible explanation is the age-dependent accumulation of the APP C-terminal fragments (CTFs) caused by a lack of *γ*-secretase activity after conditional inactivation of PS1, leading to the buildup of *γ*-secretase substrates [183]. Another explanation is the non-*γ*-secretase functions of PS1 may be involved in aspects of memory formation, storage, or consolidation, by regulating intracellular calcium dynamics. 

**Figure 4:**
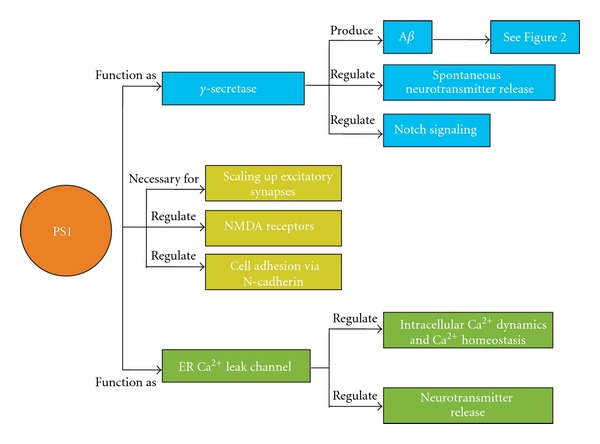
The roles of presenilins in synaptic function. Two main functions of presenilin 1 (PS1) focused on in this paper are its ability to function as part of the *γ*-secretase cleavage complex and also to function as an ER Ca^2+^ leak channel. The *γ*-secretase complex is responsible for the final cleavage of APP in the production of A*β* peptides. The *γ*-secretase complex has also been shown to regulate spontaneous neurotransmitter release and is crucial for the regulation of the notch signaling pathway especially during early development. The PS1 holoprotein has been proposed to function as an ER Ca^2+^ leak channel. It is responsible for regulating intracellular calcium dynamics and calcium homeostasis required for proper signaling and neurotransmitter release. In addition to these two main functions, knockout studies have shown that the PS1 protein is important for synaptic scaling, proper NMDAR-mediated responses, as well as cell adhesion mediated by N-cadherin. Through these studies it is clear that PS1 plays an important role in synaptic transmission and plasticity.

In addition to its proteolytic activity, PS1 is implicated in regulating neurotransmitter release [182, 245] and intracellular calcium dynamics [246–248] (Figure 4). It has been proposed that the full length PS1 can act as a passive endoplasmic reticulum (ER) Ca^2+^ leak channel [249] and that some FAD-linked PS1 mutations lack this property. However, it remains controversial [102, 250, 251] whether Ca^2+^ dysregulation that occurs during AD can be directly linked to alterations in ER Ca^2+^ leak channels formed by PS1. While the exact mechanism may be unknown, there is ample evidence that FAD-linked mutations in PS1 contribute to augmented cytosolic Ca^2+^ concentrations resulting from changes in intracellular ER Ca^2+^ dynamics [181, 246–248, 252–254]. FAD-linked mutations in PS1 appear to influence Ca^2+^ homeostasis by causing enhanced Ca^2+^ responses of ryanodine receptors (RyRs) [253–258] and inositol-1,4,5-triphosphate receptors (IP_3_Rs) [252, 259, 260] found in the ER [261], enhanced filling of ER Ca^2+^ stores [262, 263], and attenuation of capacitive Ca^2+^ entry (CCE) stores [264–267]. Presenilins have also been found to play a normal physiological role in regulating sarco-ER Ca^2+^-ATPase (SERCA) pumps that help maintain low cytosolic Ca^2+^ concentrations by pumping Ca^2+^ into ER stores [262]. SERCA activity also influences A*β* production, such that increased SERCA activity increases A*β* production [262].

Synaptic transmission and plasticity are important cellular mechanisms underlying cognitive functions, and there is evidence that presenilins play a role in these mechanisms. Mice with PS1 cKO in the cortex showed normal basal synaptic transmission, LTP, and LTD in the hippocampal Schaffer collateral pathway [178], suggesting that in the adult brain, activity of PS2 is sufficient to maintain normal synaptic properties when PS1 is absent. In contrast to PS1 cKO mice, PS1/PS2 conditional double knockout (PS cDKO) shows reduced LTP and a decreased PPF ratio at these synapses as early as 2 months of age. By 6 months, PS cDKO mice showed even greater synaptic deficits, including loss of presynaptic inputs and enhanced basal synaptic transmission, in addition to reduced LTP and PPF ratio [179]. These synaptic impairments may explain the age-dependent deterioration in the cognition of the PS cDKO mice [179]. Collectively, these studies suggest that presenilins are essential for synaptic plasticity as well as learning and memory in the adult brain. 

What is the cellular mechanism that mediates the effects of PS1 on synaptic plasticity? Saura et al. [179] found a reduction in the postsynaptic NMDAR-mediated response in PS cDKO mice, which correlated with a decrease in the cortical levels of synaptic NMDAR expression. Saura et al. also found that synaptic localization and delivery of NMDARs may depend on certain interactions with presenilins. Therefore, the downregulation of postsynaptic NMDARs is a reasonable explanation for why LTP and memory are impaired in PS cDKO mice. Loss of presenilin function also decreased the expression of both dendritic and synaptic *α*CaMKII levels as well as multiple CRE-dependent genes [179], which are all involved in the downstream signaling of NMDAR activation associated with LTP and memory formation (for a review on LTP and memory and the involved molecules, see [16]). This indicates that presenilins not only exert regulatory effects on NMDARs but also the signaling cascades that lead to LTP and memory formation. Surprisingly, later studies that looked specifically at CA1 neurons in the hippocampus revealed that, at 2 months, PS cDKO mice show an unexpected increase in the number of pre- and postsynaptic sites labeled for the NR2A subunits of NMDARs [180]. This increase is not accompanied by synapse loss or alterations in spine size, in agreement with previously documented morphology of PS cDKO mice at this age [179]. The authors [180] suggested that NMDARs become trapped at the synaptic membrane leading to excitotoxicity and eventual neurodegeneration that is present in PS cDKO mice at 6 months [179]. In addition, they suggested that LTP impairments are not due to a reduction in NMDAR number but may be more tightly linked to the reduced levels of *α*CaMKII present in the dendritic spines [180]. 

As previously mentioned, presynaptic function was also altered in PS cDKO mice: a reduced PPF ratio, which was attributed to abnormal presynaptic Ca^2+^ signaling, and a reduction in presynaptic release probability were observed [181, 182]. In addition, there was a loss of presynaptic inputs in older PS cDKO mice suggesting that certain signals necessary for maintaining axon terminals may be missing. PS1 has been found to localize at the synapse and regulate adhesive contact of pre- and postsynaptic compartments, mediated by N-cadherin [239], the major molecule that mediates Ca^2+^-dependent cell-cell interaction [268]. The diminution of N-cadherin-mediated cell-cell adhesion when presenilins are inhibited might cause the presynaptic defects in PS cDKO mice. One study sought to address the temporal progression of pre- and postsynaptic impairments in the Schaffer collateral pathway of PS cDKO mice [181]. They found that the decrease in presynaptic calcium-dependent facilitation and neurotransmitter release preceded postsynaptic impairments in NMDAR-mediated responses and LTP. Previous experiments in which presenilins were conditionally knocked out in either presynaptic, CA3 or postsynaptic, CA1 neurons [182] demonstrated that loss of presynaptic presenilin is sufficient to cause impaired glutamate neurotransmitter release and LTP, due to altered intracellular calcium signaling. However, loss of pre- or postsynaptic presenilin alone was not sufficient to cause impairments in NMDAR-mediated responses [182]. The authors propose a “trans-synaptic mechanism” to explain the alterations in postsynaptic NMDAR function [181]. In any case, presenilins are likely essential for regulating the intracellular calcium signals required for proper neurotransmitterrelease to insure normal short- and long-term plasticity. Indeed, several recent studies have found that PS1 function is important in regulating homeostatic plasticity [140] and neuronal ER Ca^2+^ homeostasis [246], as well as a novel function of the *γ*-secretase complex in regulating spontaneous neurotransmitters release [245]. Therefore, presynaptic dysfunction and altered calcium dynamics may be an early event leading to neuronal degeneration and pathogenesis in AD. 

### 8.1. Gamma-Secretase Inhibitors and Modulators

The *γ*-secretase complex is critical in the formation of A*β* peptides; hence it is one of the key therapeutic targets for stopping the progression of AD. Although many classes of compounds exist that target the *γ*-secretase complex, not many have investigated their effects on synaptic transmission and plasticity. Numerous studies have documented the ability of different classes of *γ*-secretase inhibitors (GSI) and modulators (GSM) to reduce A*β* levels in the brain [269–278], as well as their effects on cognitive function in hippocampal-dependent memory task such as the Morris water maze or contextual fear conditioning [279–282]. There are two studies [184, 185] that looked at the effects of drug treatment on synaptic plasticity in a mouse model of AD. Both studies used Tg2576 mice to investigate the ability of the GSI, MRK-560 [184], or the GSM, CHF5074 [185], to restore hippocampal memory and synaptic plasticity. Each study used different initial starting times and durations of treatment. To understand the interaction between the age-dependent increase in A*β* and its effect on basal synaptic transmission and plasticity in the CA1 region of the hippocampus, Townsend et al. [184] compared synaptic activity across three different ages, young (3-4 months), middle (6-7 months), and old (14-15 months) mice. Basal synaptic transmission was assessed by measuring the input/output activity in CA1. Even though A*β* levels continue to increase with age, the greatest synaptic deficits in Tg2576 mice were seen at 6-7 months, and in particular LTP was impaired at this middle age but was normal in both young and old mice [184]. This suggests that soluble A*β* is inversely correlated with LTP, until plaque deposition occurs, when soluble A*β* can no longer predict LTP impairments [184]. Since 6-7-month-old mice showed the greatest deficits, they were given oral doses for 1, 3, or 7 days with the GSI, MRK-560. After 1 day, A*β* levels were significantly reduced and LTP began to improve. LTP improvements reached significance after 3 days of dosing. After 7 days of treatment, basal synaptic transmission began to recover but did not reach significance. This supports the theory that lowering A*β* levels can recover synaptic plasticity in 6-7-month-old Tg2576 mice, before plaque deposition. Balducci et al. [185] also focused on how the GSM, CHF5074 may be able to rescue synaptic deficits seen in plaque-free Tg2576 mice. After acute subcutaneous treatment with CHF5074, 5-month-old Tg2576 mice showed significantly reduced contextual memory impairments [185]. At 6 months old, after receiving a 4-week subchronic oral treatment, which reduced intraneuronal A*β* level, the impairments in recognition memory and hippocampal LTP were reversed. To determine if aged mice would also show improvements after treatment, daily doses of MRK-560 were given to Tg2576 mice from 12–15 months of age [184]. Since LTP was similar to wildtypes at this age, the focus was on basal synaptic transmission. Similar to middle-aged animals, treatment with MRK-560 significantly reduced A*β* levels; however, there was no improvement in basal synaptic transmission. The lack of functional recovery in older age group was also seen in APP Tg mice crossed with PS1 cKOs [183]. These results reveal that even though conditional inactivation of PS1 can successfully reduce A*β* production and the amyloid-associated neuropathological alterations, it does not prevent the impairments in both synaptic and cognitive functions [183]. Collectively, these studies suggest that the effects of A*β* on basal synaptic transmission and plasticity differ with age and that successful reduction of A*β* levels by targeting APP-processing enzymes may not recover synaptic dysfunctions.

## 9. Conclusion

It is clear that successful AD treatments will need to do more than just lower A*β* production; they will need to rescue cognitive as well as synaptic dysfunctions. Increasing evidence suggests the cognitive syndromes found in AD patients are preceded by changes in synaptic efficacy (reviewed in [1, 283]). Therefore, examining whether different strategies that target APP-processing enzymes rescue synaptic dysfunctions associated with AD is important. Several current reviews state why certain APP-processing drug therapies have failed in recent clinical trials and why current trials have not been able to generate more beneficial or significant results [284–286]. Testing the effects of potential AD therapeutics on synaptic function, in addition to behavioral analyses, will provide a better mechanistic understanding of the potential problems. It is also important to remember how different animal models may affect the outcome of results. For example, in mouse studies, genetic background has been shown to influence the effectiveness of certain *γ*-secretase targeting drugs [287–289]. In addition, many AD mouse models have been generated from FAD-linked mutations and may not fully recapitulate sporadic AD cases. In sum, mechanistic understanding of the normal synaptic functions of APP-processing enzymes will benefit the development of more effective treatments for AD.

## Figures and Tables

**Table 1:** Summary of known synaptic effects of altering BACE1.

	Age	A*β*	Basal synaptic transmission	Presynaptic function	LTP	LTD	Reference
BACE1 KO	3–6 mo	No A*β*_40_ or A*β*_42_	Normal (CA1 and CA3)	Increased PPF ratio (CA1 and CA3)	Normal LTP (4x TBS) but larger dedepression in CA1; no mossy fiber LTP (3x 100 Hz) and no dedepression in CA3	Normal LTD (paired-pulse 1 Hz) in CA1, but slightly larger LTD (paired-pulse 1 Hz) in CA3	[164, 174]

BACE1 KO + activation of *α*7-nAChRs	3–6 mo	No A*β*_40_ or A*β*_42_	Normal (CA3)	Restored PPF ratio (CA3)	Rescued mossy fiber LTP (CA3) (3x 100 Hz)		[175]

BACE1^+/−^; 5XFAD APP/PS1 (Tg6799)	6 mo	66% decrease in A*β*_40_ and 57% in A*β*_42_ in brain; reduce amyloid plaque burden in hippocampus by 78% and anterior cingulate cortex by 44%	Remained significantly reduced (CA1)		Restored LTP to WT control levels (CA1) (3x TBS)		[176]

Adenoviral-Fbx2^∗^ in Tg2576	12–14 mo	30% decrease in A*β*_42_	No change		Improved the impaired LTP (CA1) (3x TBS) 4 weeks after adenoviral injection		[177]

^∗^Transfected into hippocampus. Fbx2 facilitates BACE1 degradation.

**Table 2:** Summary of alterations in synaptic function by altering presenilin or *γ*-secretase activity.

	Age	A*β*	Basal synaptic transmission	Presynaptic function	LTP	LTD	Other	Reference
PS1 cKO	3–6 mo	Cortical A*β*_40_ and A*β*_42_ are decreased	Normal (CA1)	Normal PPF ratio (CA1)	Normal (CA1) (5x TBS) normal (CA1) (3x 100 Hz)	Normal (CA1)		[178]

	2 mo		Normal (CA1)	Decreased PPF ratio (CA1)	Decreased (CA1) (5x TBS)	Normal (CA1)	Reduced NMDAR function (CA1); decreased cortical synaptic levels of NR1, NR2A, *α*CaMKII, and CRE-dependent genes	[179]
	6 mo		Increased (CA1)	Decreased PPF ratio (CA1)	Decreased (CA1) (5x TBS)	Normal (CA1)		
PS cDKO	2 mo						Increased expression of the NR2A subunit of NMDARs, specifically at postsynaptic density and presynaptic terminals of axo/dendritic synapses, trapped at synapses (CA1)	[180]
	5 wk			Decreased Ca^2+^-dependent frequency facilitation and release probability (CA1)	Normal (CA1) (5x TBS)		Normal NMDAR-mediated synaptic response (CA1)	[181]
	6 wk				Decreased (CA1) 5x TBS		Reduced NMDAR-mediated synaptic response (CA1)	

CA1-PS cDKO	2 mo			Normal (CA1)	Normal (CA1) (5x TBS)		Normal NMDAR/AMPAR ratio	[182]

CA3-PS cDKO	2 mo			Decreased Ca^2+^-dependent frequency facilitation and release probability (CA1)	Decreased (CA1) (5x TBS)		Normal NMDAR/AMPAR ratio	[182]

AppTg (Swe/Ind)	3 mo		Normal (CA1)	Normal (CA1)	Increased (CA1) (5x TBS)			[183]
	6 mo	Age dependent increase in A*β* levels and plaque deposition	Decreased (CA1)	Normal (CA1)	Decreased (CA1) (5x TBS)			

AppTg; PS1^−/−^	3 mo		Normal (CA1)	Normal (CA1)	Increased (CA1)			[183]
	6 mo	Decreased cortical A*β* peptides and plaque formation	Decreased (CA1)	Normal (CA1)	Decreased (CA1) (5x TBS)			

	3-4 mo		Normal (CA1)		Normal (CA1)			
Tg2576	6-7 mo	50% increase in soluble A*β*	Decreased (CA1)-stronger stimulus required to elicit similar sized postsynaptic responses		Decreased (CA1)			[184]
	14-15 mo	1000% increase in soluble A*β*	Decreased (CA1) but similar to 6-7 months		Normal (CA1)			

Tg2576 w/MRK-560 (*γ*-secretase inhibitor)	6-7 mo	1, 3, or 7 days of treatment reduced soluble A*β* levels	Partial recovery but not significant		Recovered (CA1)			[184]
	15 mo	3 months of treatment reduced soluble A*β* levels	No improvement					

Tg2576 w/CHF5074 (*γ*-secretase modulator)	6 mo	4-week, subchronic, oral treatment reduced A*β* levels			Recovered (CA1)			[185]
